# Ileocolonic anastomosis after right hemicolectomy for colon cancer: functional end-to-end or end-to-side?

**DOI:** 10.1186/1477-7819-12-306

**Published:** 2014-10-07

**Authors:** Zheng Liu, Guiyu Wang, Ming Yang, Yinggang Chen, Dazhuang Miao, Shan Muhammad, Xishan Wang

**Affiliations:** Department of Colorectal Surgery, The Second Affiliated Hospital of Harbin Medical University, 246 Xuefu Road, 150081 Harbin, China; Department of Gastrointestinal Medical Oncology, The Third Affiliated Hospital of Harbin Medical University, 150 Haping Road, 150040 Harbin, China; Colorectal Cancer Institute, Harbin Medical University, 246 Xuefu Road, 150081 Harbin, China

**Keywords:** anastomosis, colon cancer, right hemicolectomy

## Abstract

**Background:**

The purpose of this study was to compare short-term clinical outcomes of ileocolonic functional end-to-end anastomosis (FEEA) and end-to-side anastomosis (ESA) following resection of the right colon for cancer.

**Methods:**

We enrolled 379 patients who underwent ileocolonic anastomosis following resection of the right colon for cancer by a single surgeon, from January 2009 through June 2012. Patient characteristics, operative results, and postoperative complications were analyzed.

**Results:**

A total of 164 patients received ESA and 215 patients received FEEA. The FEEA group had a lower incidence of anastomotic error (0.9% versus 4.3%; *P* = 0.04) and a shorter operating time (140.4 ± 14.9 min versus 150.5 ± 20.1 min; *P* = 0.001). The length of hospital stay (10.9 ± 3.5 days versus 11.3 ± 4.0 days; *P* = 0.36) and anastomotic leakage (1.8% versus 0.5%; *P* = 0.20) were similar in both groups. No relevant differences between FEEA and ESA were observed for blood loss, retrieved lymph nodes, first flatus and postoperative complications.

**Conclusion:**

An FEEA after right hemicolectomy for colon cancer is a safe and reliable anastomotic technique, resulting in a favorable outcome in selected patients with the right colon cancer.

## Background

Performing anastomosis after colectomy is one of the basic skills of a general surgeon [1]. Bowel anastomosis is conventionally performed using a handsewn technique, which has been practiced successfully for over 100 years [2]. Because stapled anastomosis takes less time to perform and the learning curve for the inexperienced surgeon is short, mechanical stapling devices are widely used in gastrointestinal surgery [3–5].

There are several configurations of ileocolonic anastomosis, such as functional end-to-end anastomosis (FEEA) and end-to-side anastomosis (ESA) [6]. The end-to-end anastomosis (EEA) is possible only using the handsewn technique. An ESA is commonly used in a right hemicolectomy. This anastomosis proceeds in a manner very similar to that of the EEA [7].

Because of the disparity in size between the ileum and colon, the anastomotic complications of ESA and EEA are not rare [8]. In an effort to decrease anastomotic complications, the stapled FEEA was developed [9]. The FEEA is a side-to-side anastomosis, and follows the excision of the ileocecal site and the ascending colon using linear staplers. There are many theories to explain why FEEA should fare better, including the wider diameter, a reduction in intraluminal pressure, and less proximal ischemia [10]. The literature seems to suggest that the FEEA has become the most commonly preferred technique in recent times [11].

Although a stapled anastomosis is generally recommended, the clinical outcomes of FEEA and ESA have not been investigated sufficiently. There are no studies specifically comparing FEEA and ESA after right hemicolectomy for colon cancer. The aim of this retrospective study was to compare the complications and effects of FEEA and ESA after right hemicolectomy for colon cancer.

## Methods

From January 2009 to June 2012, 379 patients who received a right hemicolectomy by open surgery for tumors in the cecum, ascending colon, of transverse colon were included in this study. The choice between the two anastomoses was left to the surgeon. All operations were performed by the same colorectal surgeon in our hospital. The surgeon had more than 20 years of experience in open colorectal surgery and had performed more than 400 open colorectal surgeries per year for the previous 3 years.

The diagnosis of colon cancer was confirmed by a thorough physical examination and preoperative investigation. Preoperative investigation included a complete colonoscopy with biopsy, chest X-ray, relevant serum tests, and ultrasonography or computed tomography of the abdomen. The study was approved by the ethics committee of the Second Affiliated Hospital of Harbin Medical University. Patients with acute intestinal obstruction, recurrent opening of the abdominal cavity, current immunosuppressive therapy, distant metastasis, locally advanced cancer, or severe psychiatric or neurologic diseases were excluded from this study. All patients were given preoperative antibiotic prophylaxis with 2 g cefotiam and 0.5 g metronidazole.

Patient-related factors that were recorded were; age, sex, body mass index, and American Society of Anesthesiologists classification. Operation-related factors that were recorded were operating time, blood loss, number of retrieved lymph nodes, length of hospital stay, positive resection margin, time to first flatus, and complications.

**Figure 1:**
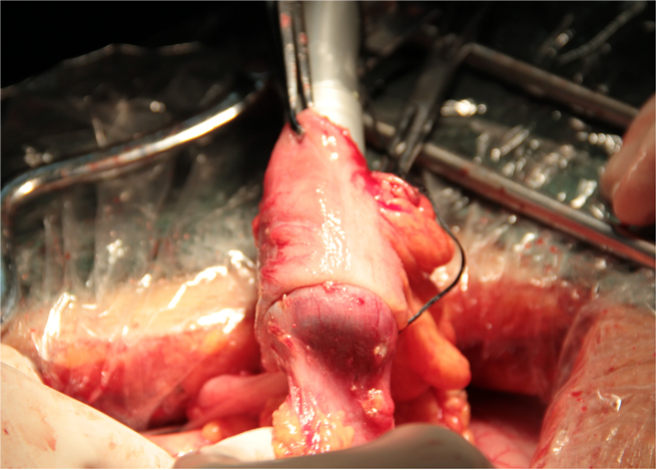
**End-to-side anastomosis: withdrawal of circular stapler.**

**Figure 2:**
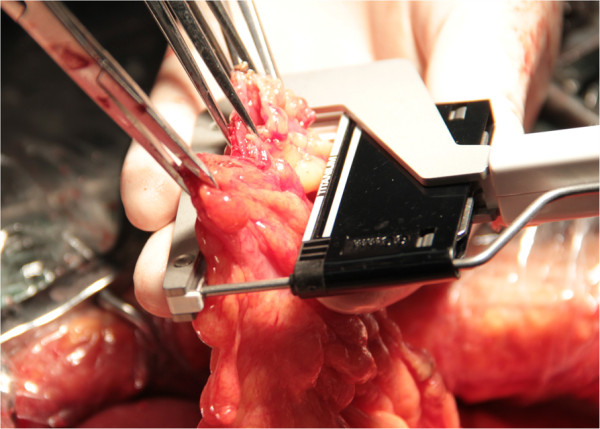
**End-to-side anastomosis: closure of colon by linear stapler.**

The operation is initiated in the standard fashion. The right colon is mobilized, the sites of division of the transverse colon and ileum are selected, and the mesentery is divided. For the ESA maneuver, the anvil of a circular stapler (Proximate, CDH29; Ethicon Endo-Surgery, Cincinnati, Ohio) is positioned in the lumen of the distal ileum using a purse string suture. The device is inserted through the open end of the colon. The trocar must pierce through one of the teniae, the aim being to select the proper antimesenteric orientation for the colonic end of the anastomosis. The trocar and anvil can then be connected; the instrument is now closed, fired, opened and carefully withdrawn (Figure 1). The open end of the colon is now closed by the linear stapler (Proximate, TLC55; Ethicon Endo-Surgery, Cincinnati, Ohio) (Figure 2).

**Figure 3:**
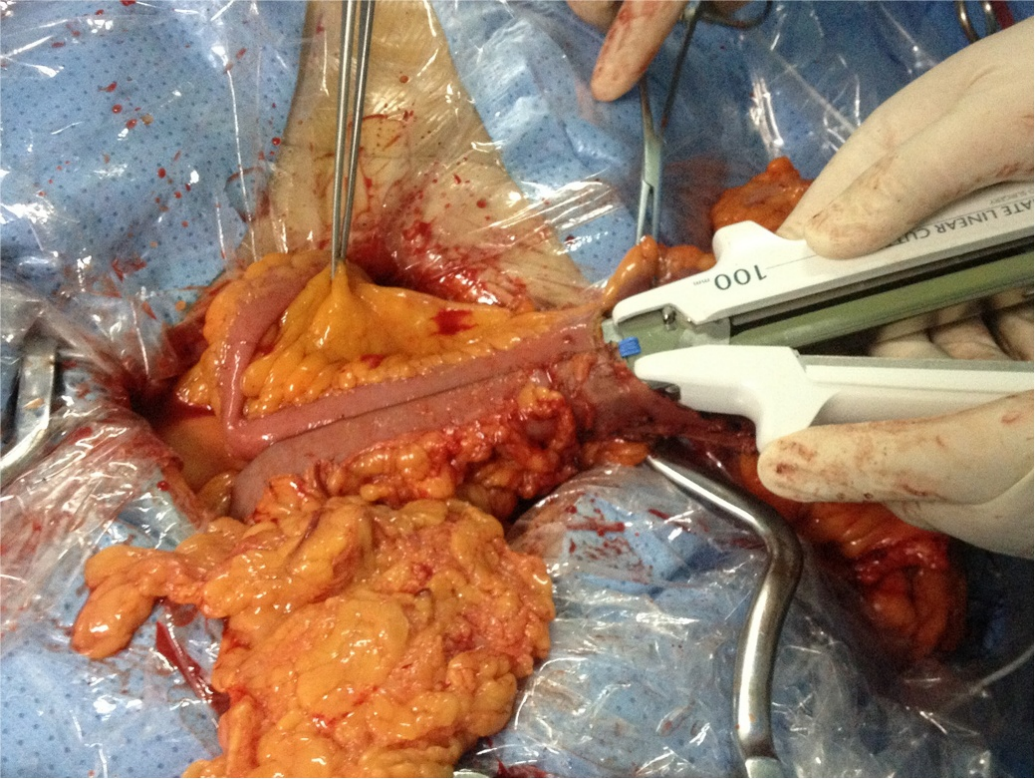
**Functional end-to-end anastomosis: firing of stapler to produce anastomosis.**

**Figure 4:**
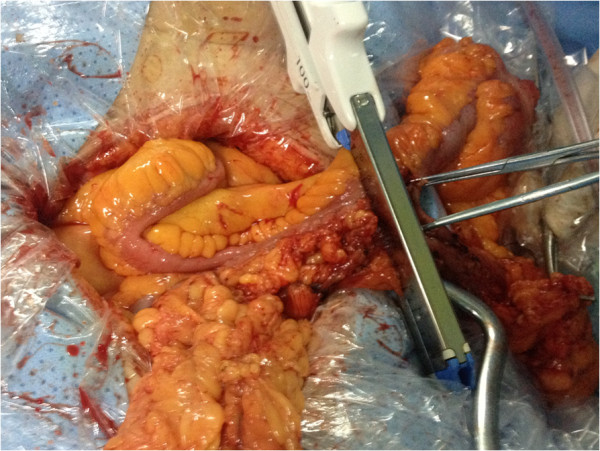
**Functional end-to-end anastomosis: resection of ileocecal site using stapler.**

For the FEEA maneuver, small holes were made in the walls of the ileum and the colon using an electric scalpel. The prongs of the linear stapler (Proximate, TLC75 or TLC10; Ethicon Endo-Surgery, Cincinnati, Ohio) were inserted in these holes and were fired to perform the anastomosis (Figure 3). We waited for 30 s before releasing the stapler to allow for hemostasis. The mucosal lumen of the anastomosis was then examined carefully for hemostasis and any bleeding points were hemostated with 4–0 coated vicryl (VCP771D; Ethicon, Somerville, NJ, USA). The ileocecal site, which contained the tumor and the holes, was resected using the same stapler (Figure 4). The stapler edge was also carefully observed, and any bleeding points were hemostated with 4–0 coated vicryl. The crotch of the side-to-side anastomosis was buttressed by three stitches of 4–0 coated vicryl. The gap in the mesentery is repaired and two drains are left in the right subhepatic area. The abdomen is closed in layers in the standard way.

### Statistical analysis

Values are means ± standard deviations or numbers (percentages). Statistical analysis was performed using SPSS statistical software for Windows Version 13.0 (SPSS, Chicago, IL, USA). Quantitative parametric data were compared between groups using the *t* test, and nonparametric data were compared using the Mann–Whitney test. Qualitative data were compared using the chi-square and Fisher’s exact tests. A *P* value of 0.05 or less was considered to be statistically significant.

## Results

The characteristics of the patients are described in Table 1. In all, 379 patients were enrolled for participation in this study. Of these, 164 patients received ESA and 215 patients received FEEA. There were no significant differences between the ESA and the FEEA groups in terms of age, sex, body mass index, American Society of Anesthesiologists classification, and ‘tumor, node, metastases’ (TNM) classification.

**Table 1:** **Clinicopathologic characteristics of patients**

Variable	End-to-side anastomosis (***n***= 164)	Functional end-to-end anastomosis (***n***= 215)	***P***
Age (years)	63.2 ± 10.1	62.5 ± 9.0	0.49
Sex:			0.87
Male	96 (58.5%)	124 (57.7%)	
Female	68 (41.5%)	91 (42.3%)	
Body mass index (kg/m^2^)	21.8 ± 2.6	22.0 ± 2.9	0.40
American Society of Anesthesiologists classification:			0.66
1	67	83	
≥2	97	132	
TNM classification			0.15
Stage I	12	10	
Stage II	85	97	
Stage III	67	108	

Data presented as mean ± standard deviation or *n* (%). TNM, ‘tumor, node, metastases’.

The operative results are summarized in Table 2. Blood loss tended to be somewhat less in the ESA group than in the FEEA group, although the difference was not statistically significant (93.1 ± 29.4 ml versus 100.2 ± 40.0 ml; *P* = 0.05). A significantly shorter operating time was observed in the FEEA group than in the ESA group (140.4 ± 14.9 min versus 150.5 ± 20.1 min; *P* = 0.001). An average of 14.8 ± 7.3 lymph nodes were harvested in the ESA group, versus 15.9 ± 9.4 in the FEEA group (*P* = 0.22). There were no significant differences in time to first flatus or the length of hospital stay. Seven cases (4.3%) involved ESA error and two cases (0.9%) involved FEEA error.

**Table 2:** **Operative results**

Variable	End-to-side anastomosis (***n***= 164)	Functional end-to-end anastomosis (***n***= 215)	***P***
Operating time (min)	150.5 ± 20.1	140.4 ± 14.9	0.001
Blood loss (ml)	93.1 ± 29.4	100.2 ± 40.0	0.05
Retrieved lymph nodes	14.8 ± 7.3	15.9 ± 9.4	0.22
Anastomotic error	7 (4.3%)	2 (0.9%)	0.04
Positive resection margin	0	0	
First flatus (d)	2.7 ± 0.9	2.5 ± 1.3	0.18
Length of hospital stay (d)	10.9 ± 3.5	11.3 ± 4.0	0.36

Data presented as mean ± standard deviation or *n* (%).

Postoperative complications are described in Table 3. The rates of postoperative complication did not differ significantly between groups. There was no abscess and no death in any group. One patient who received FEEA had a bowel obstruction. She underwent another operation. The overall incidence of anastomotic leakage was 1.1% (4/379). Anastomotic leakage after ESA was 1.8% (3/164), while that after FEEA was 0.5% (1/215). All of these patients were successfully treated with local washouts and antibiotics.

**Table 3:** **Postoperative complications**

Variable	End-to-side anastomosis (***n***= 164)	Functional end-to-end anastomosis (***n***= 215)	***P***
Wound infection	2 (1.2%)	2 (0.9%)	0.79
Bowel obstruction	1 (0.6%)	2 (0.9%)	0.73
Anastomotic leakage	3 (1.8%)	1 (0.5%)	0.20
Haemorrhage	3 (1.8%)	2 (0.9%)	0.45
Abscess	0	0	
Urinary tract infection	3 (1.8%)	2 (0.9%)	0.45
Pneumonia	1 (0.6%)	3 (1.4%)	0.46
Secondary surgery	1 (0.6%)	0	0.43
Death	0	0	
Others	3 (1.8%)	5 (2.3%)	0.74

Data presented as mean ± standard deviation or *n* (%).

## Discussion

The anastomotic technique selected for colectomy depends upon the site of cancer, bowel diameter, and surgeon’s personal experience [12–14]. Stapled anastomosis is now widely accepted in right hemicolectomy because the stapling procedure is simple, reliable, and safe [15]. In the literature, there are many studies comparing stapled and handsewn anastomoses [16–18]. However, there is no study that compares ESA and FEEA in right hemicolectomy for colon cancer. In this study, we have demonstrated that the FEEA is a safe and timesaving procedure.

Steichen suggested in 1968 [9] that the FEEA simplifies and hastens creation of the anastomosis and overcomes anastomotic stricture. At present, a standard right hemicolectomy using a stapling procedure is not a difficult procedure. The number of FEEAs performed is now increasing [19]. We did not find clear evidence in the literature that anastomotic configuration *per se* could influence the risk of leakage. Because of its wider diameter and superior blood supply, FEEA may reduce intraluminal pressure and proximal ischemia [20]. The rates of anastomotic leakage following FEEA have been reported to range from 0 to 7.1% [21–23]. A recent retrospective, multicentric study comparing the incidence of anastomotic leakage in ileocolonic anastomosis showed ESA to be superior to FEEA [16]. The results of the present study showed that there were no differences between FEEA and ESA in anastomotic leakage rates; these findings conform to a previous meta-analysis, although there was a lower incidence in the FEEA group [24].

Conversely, the FEEA is criticized, because it has long anastomotic lines, and it is thought that it may have a higher rate of complications than other techniques [16]. All stapled procedures have an inherent risk of anastomotic errors, which is independent of the devices used. Inspection post-deployment involved a visual assessment of the anastomotic donuts, air leak testing, and endoscopic examination of the staple line. Those errors identified on post-deployment inspection were considered anastomotic errors [25]. Circular stapling techniques are also associated with intraoperative mishaps in up to 10% of cases [26]. Anastomotic error involved operator error, staple line defects, incomplete donuts, and primary device failure. Offodile *et al.* [25] reported that the incidence of circular stapler device technical error was 19%. In our series, the more common error types for the ESA group were anastomotic bleeding and inadequate donuts. The error types for the FEEA group were anastomotic bleeding from the line of anastomosis. Regarding this, a check should always be carried out and any bleeding point secured with a suture, before completing the anastomosis.

It is commonly recognized that FEEA is an easy and timesaving technique. In 2007, the Cochrane Collaboration published a meta-analysis of randomized controlled trials regarding ileocolonic anastomoses; their findings recommended a FEEA following a right hemicolectomy, particularly if this operation is performed for a colon cancer [27]. In our study, the operating time was significantly shorter in the FEEA group than in the ESA group. The actual time required for anastomosis should be shorter in the FEEA group than in the ESA group. Although anastomotic configuration is considered a relevant factor of complications, the overall incidence of complications associated with both groups is minimal, showing that FEEA is safe. Moreover, emergency colectomies were not included, as they are obviously associated with higher infective and anastomotic failure rates.

## Conclusions

Despite many studies in the literature, the best type of anastomosis right hemicolectomy for colon cancer remains an unresolved issue. According to the results of our study, we recommend construction of an ileocolonic anastomosis with FEEA for right hemicolectomy. Further prospective investigation is required.

## Consent

Written informed consent was obtained from the patients for the publication of this report and any accompanying images.

## Abbreviations

EEA: end-to-end anastomosis
ESA: end-to-side anastomosis
FEEA: functional end-to-end anastomosis
TNM: ‘tumor, node, metastases’.
